# Social Vulnerability and Subclinical Cardiovascular Risk in Obstructive Sleep Apnea

**DOI:** 10.21203/rs.3.rs-8894664/v1

**Published:** 2026-03-18

**Authors:** Ahana Gupta, Ariana Gavanescu, Abdelhamid Ghanem, Bharati Prasad

**Affiliations:** University of Illinois Chicago; University of Illinois Chicago; University of Illinois Chicago; University of Illinois Chicago

## Abstract

Obstructive sleep apnea (OSA) is an established risk factor for cardiovascular disease (CVD). Multiple clinical factors, including OSA severity, obesity, smoking, and hypertension, mediate this risk. However, whether the association between OSA and CVD is influenced by non-clinical factors, specifically social determinants of health, remains unclear. We examined the association between neighborhood-level social determinants of health, indicated by the Social Vulnerability Index, and subclinical CVD risk in adults with untreated severe OSA. Racial and ethnic minority status (REMS) and socioeconomic status (SES) social vulnerability themes were associated with nocturnal hypertension and central arterial stiffness, independent of clinical factors. These findings highlight the need to investigate and integrate social determinants of health in OSA management and research to mitigate CVD risk.

## Introduction

Obstructive sleep apnea (OSA) and cardiovascular disease (CVD) share well-established clinical risk factors, such as obesity and smoking. However, fewer studies have examined how non-clinical risk factors, such as social vulnerability, contribute to CVD risk in adults with OSA. The Social Vulnerability Index (SVI), developed by the Centers for Disease Control and Prevention, is a comprehensive measure of neighborhood-level social determinants of health (SDOH).^1^ It encompasses four core themes: socioeconomic status (SES), household characteristics (HC), racial and ethnic minority status (REMS), and housing type and transportation (HTT). These themes, derived from sixteen factors, are scored on a scale of 0 to 1, with higher values indicating greater vulnerability. The SES subtheme includes six variables (below 150% poverty, unemployed, housing cost burden, no high school diploma, no health insurance, and per capita income), HC subtheme includes five variables (age ≥ 65 and ≤ 17 years, civilian with disability, single-parent households, and limited English proficiency), REMS subtheme includes one variable (percentage in racial/ethnic minority groups), and HTT includes four variables (multi-unit structures, mobile homes, crowding, no vehicle, and group quarters).^2^ While individual themes have demonstrated distinct associations with CVD outcomes, the evidence remains limited regarding their role in increasing subclinical CVD risk, which provides a window for preventive care.^2^

To address this gap, we investigated the impact of neighborhood-level vulnerability, as captured by SVI themes, on subclinical CVD risk in an urban population with severe, untreated OSA. We hypothesized that higher vulnerability in one or more SVI themes would be associated with greater subclinical CVD risk.

## Methods

### Participants and Procedures

Sixty adults aged 18 to 65 years with newly diagnosed moderate to severe OSA (polysomnography apnea-hypopnea index using the 3% criterion; AHI > 15/hour) were recruited for this observational study. All participants provided informed consent, and demographic and clinical data were recorded. Participants completed a single study visit including overnight polysomnography (9 PM to 5 AM) with beat-to-beat blood pressure monitoring (8 PM to 5 AM with Finapres^®^ NOVA), followed by fasting morning blood sampling and vascular function assessments (Augmentation Index; **AIx** and carotid-femoral pulse wave velocity; **PWV** with SphygmoCor XCEL and Vascular Reactivity Index; **VRI** with VENDYS II). Informed consent was obtained from participants per ethical board regulations. The University of Illinois Chicago Institutional Review Board (IRB) approved the study (IRB number: STUDY2022–1039-MOD001). All study procedures were performed in accordance with the IRB guidelines and regulations.

### Subclinical CVD Risk Indicators (Outcomes)

Beat-to-beat blood pressure and polysomnography data were used to determine OSA severity and calculate the average mean arterial pressure during sleep (**MAPs**). OSA severity was also assessed using hypoxia burden (HB), which is more strongly associated with CVD compared to AHI.^3, 4^ AIx and PWV were used as indicators of arterial stiffness, and endothelial function was quantified by the VRI. Fasting blood samples were assayed for high-sensitivity C-reactive protein (**hsCRP**, systemic inflammation), Homeostatic Model Assessment for Insulin Resistance (**HOMA-IR**), and plasma norepinephrine (sympathetic activation).

### Statistical Analyses

Baseline clinical characteristics were summarized using descriptive statistics. The distribution of outcomes was examined with histograms and Q-Q plots. HOMA-IR, hsCRP, norepinephrine, and AIx were log-transformed to achieve normality. Multivariable linear regression was conducted, with each SVI theme as a predictor of subclinical CVD risk indicators. The models were adjusted for the following covariates: age, sex, body mass index (BMI), smoking, number of antihypertensive medications, and AHI. For models where an SVI theme was a significant predictor of a subclinical CVD risk indicator, sensitivity analyses were conducted by substituting AHI with HB as an OSA severity indicator. A two-sided p-value of < 0.05 was considered significant without multiple comparisons correction. STATA v17 was used for analyses.

## Results

The demographic characteristics of the participants are shown in Table 1. The sample consisted of middle-aged individuals, with the majority being men, and African Americans or Hispanics. OSA was severe with clinically significant sleepiness, impaired sleep quality, and an overall SVI in the highest quartile, reflecting high social vulnerability. The plasma assays for inflammation (hsCRP), insulin resistance (HOMA-IR), and sympathetic activation (norepinephrine) were not associated with SVI themes (Table 2). As expected, high hsCRP and HOMA-IR were significantly associated with BMI, and a higher norepinephrine level was significantly associated with both OSA severity indicators: AHI and HB. VRI (endothelial function) and AIx (a measure of central and peripheral arterial stiffness) were not associated with SVI themes but were associated with BMI. AIx also significantly increased with age (data not shown).

As shown in Table 2, MAPs was significantly associated with the SVI theme of REMS (t = 3.34, p = 0.002) with a large effect size (eta squared; *η*^2^ = 0.18; 95% confidence interval, CI 0.03–0.33). Similar results were observed when the model was adjusted for HB instead of AHI (t = 3.25, p = 0.002). PWV is considered a gold-standard measure of central arterial stiffness.^5^ PWV was significantly associated with the SVI theme of SES (t = 2.22, p = 0.03 for AHI model and t = 3.41, p = 0.01 for HB model), and a medium effect size was noted (*η*^2^ = 0.10, 95% CI 0.01–0.28). For MAPs and PWV, other covariates were not significant, including BMI and AHI or HB.

## Discussion

This study demonstrates that higher social vulnerability (SVI) is associated with increased subclinical CVD risk in adults with severe OSA. Specifically, higher REMS vulnerability was associated with elevated MAPs, indicating nocturnal hypertension, and higher SES vulnerability was associated with increased central arterial stiffness. These findings underscore the importance of incorporating neighborhood-level SDOH into the development and implementation of clinical and community-level CVD risk-mitigation interventions.

Our finding that REMS vulnerability is associated with nocturnal hypertension aligns with prior work showing elevated risk among African American and Hispanic individuals.^6, 7^ Although OSA is a known risk factor for nocturnal hypertension, we did not observe AHI or HB as significant predictors, which differs from previous findings.^8^ This discrepancy is likely due to differences in the study population and methodologies (clinical vs. community, OSA severity, or methods of blood pressure monitoring). Lower neighborhood-level SES has been associated with higher PWV in community samples,^9^ but evidence in high-risk clinical populations remains limited. Our results suggest that neighborhood-level SES vulnerability increases arterial stiffness and subclinical CVD risk independent of clinical factors such as OSA severity and obesity. Previous studies have reported conflicting results regarding the effect of AHI on carotid-femoral PWV, likely due to differences in study populations and designs.^10–12^ In this study, the absence of associations between OSA severity (AHI or HB) or obesity and MAPs or PWV may reflect a ceiling effect, driven by the predominance of severe OSA and class II obesity. In contrast, AHI increased sympathetic activity, and BMI was associated with higher inflammation and insulin resistance in the fully adjusted models. These data suggest that OSA severity, obesity, and SDOH may affect CVD risk through different cardiometabolic pathways.

This study extends growing evidence that neighborhood-level SDOH function as upstream determinants of CVD risk in OSA.^13, 14^ Neighborhood-level social vulnerability has been shown to increase the risk and severity of OSA.^15^ Further, SVI has demonstrated utility in capturing SDOH relevant to CVD risk and other crucial factors that affect CVD outcomes such as access to preventive care and healthy foods.^2^ Emerging evidence indicates that targeted interventions in underserved populations improve the effectiveness of OSA management.^16^ Our results highlight the need for empirical testing of targeted OSA interventions on CVD outcomes in at-risk populations identified by high SVI, as well as the potential value of policy-level efforts to improve equitable access to OSA care.

Notable strengths of this study include the use of a validated assessment of neighborhood-level social vulnerability, a comprehensive evaluation of subclinical CVD risk indicators, gold-standard measurements of OSA severity and arterial stiffness,^17^ and cuffless, beat-to-beat blood pressure monitoring that is recommended for nocturnal hypertension assessment.^18^ However, some limitations warrant consideration. First, individual-level SDOH and their potential interactions with neighborhood-level vulnerability were not assessed. Second, the limited urban clinical sample, characterized by severe OSA and high burden of obesity, may limit generalizability to individuals with milder OSA or those residing in rural settings. Thus, the results of this cross-sectional study may be considered hypothesis-generating, and the association of specific subthemes with CVD risk factors and effect size estimates can be used to guide future research in OSA. Future work should examine multi-level SDOH and broader populations in adequately powered longitudinal studies to inform CVD prevention and policy.

## Figures and Tables

**Table 1:** Baseline Characteristics

Age (years)	44.9 ± 9.4

Sex (male)	41 (68.3)

Race/Ethnicity	5 (8.3)
White	37 (61.7)
African American	16 (26.7)
Hispanic	2 (3.3)
Other	

Social Vulnerability Index	0.76 ± 0.17

Body Mass Index	37.1 ± 6.99

Smoking (current)	12 (20)

Resting systolic BP, mmHg	131.4 ± 18.4

Resting diastolic BP, mmHg	83.9 ± 15.9

Apnea Hypopnea Index (3%)	69.4 ± 40.7

Sleep time < 88% (minutes)	40.2 ± 63.5

Epworth Sleepiness Scale	11.6 ± 5.9

Pittsburgh Sleep Quality Index	9.5 ± 3.9

Functional Outcomes of Sleep	15.5 ± 3.4

Diabetes	12 (21.6)

Stroke	4 (6.6)

Heart Disease	3 (5)

Hypertension	29 (48.3)

Number of antihypertensives	0.8 ± 1.1

N = 60, Data are presented as mean ± standard deviation or frequency (%).

* 27 participants were on antihypertensive medications. Stroke and heart disease were self-reported but not confirmed on an electronic health records review.

**Table 2:** Association of Subclinical CVD Risk Markers with SVI Subthemes

Outcomes	Socioeconomic Status		Household Characteristics		Racial & Ethnic Minority Status		Housing Type & Transportation	
	T-stat	p-value	T-stat	p-value	T-stat	p-value	T-stat	p-value
hsCRP	−1.18	0.24	−1.06	0.29	0.51	0.61	0.88	0.38
HOMA-IR	1.53	0.13	0.35	0.73	1.76	0.08	−0.05	0.96
Plasma Norepinephrine	0.08	0.93	0.15	0.88	0.70	0.48	0.47	0.64
Mean Arterial BP in Sleep	1.77	0.08	1.12	0.27	**3.34** *	**0.002**	0.93	0.35
Vascular Reactivity Index	0.32	0.75	−0.32	0.75	0.79	0.43	−0.63	0.53
Augmentation Index	1.51	0.13	1.08	0.28	0.01	0.99	0.06	0.95
Pulse Wave Velocity	**2.22** **	**0.03**	−1.30	0.19	0.18	0.85	0.30	0.76

**Key**: CVD=cardiovascular disease, SVI=social vulnerability index, hsCRP=high sensitivity c-reactive protein, HOMA-IR=homeostatic model assessment of insulin resistance, BP=blood pressure. T-stat = T statistic in multivariable regression; all models were adjusted for age, sex, body mass index, current smoking status (yes/no), number of antihypertension medications, and apnea hypopnea index (AHI). In models where an SVI theme was a significant predictor, sensitivity analyses were conducted by replacing AHI with hypoxia burden.

* Eta-squared, *η*^2^ = 0.18 (95% confidence interval 0.03–0.33).

** Eta-squared, *η*^2^ = 0.10 (95% confidence interval 0.01–0.28).

## Data Availability

Yes
